# Predictive factors for high-flow nasal cannula failure in patients with acute viral bronchiolitis admitted to the pediatric intensive care unit

**DOI:** 10.62675/2965-2774.20250161

**Published:** 2025-02-10

**Authors:** Patrick Jacobsen Westphal, Cassiano Teixeira, João Ronaldo Mafalda Krauzer, Mirelle Hugo Bueno, Priscilla Alves Pereira, Sandro V. Hostyn, Marcela Doebber Vieira, Camila Durante, Cristiane Bündchen

**Affiliations:** 1 Hospital Moinhos de Vento Porto Alegre RS Brazil Hospital Moinhos de Vento - Porto Alegre (RS), Brazil.; 2 Universidade Federal de Ciências da Saúde de Porto Alegre Porto Alegre RS Brazil Universidade Federal de Ciências da Saúde de Porto Alegre - Porto Alegre (RS), Brazil.

**Keywords:** Bronchiolitis, High-flow nasal cannula, Treatment failure, Oxygen therapy, Pediatric intensive care unit, Respiratory insufficiency

## Abstract

**Objective:**

To identify predictive factors for failure in the installation of high-flow nasal cannulas in children diagnosed with acute viral bronchiolitis under 24 months of age admitted to the pediatric intensive care unit.

**Methods:**

This work was a retrospective single-center cohort study conducted from March 2018 to July 2023 involving infants under 24 months of age who were diagnosed with acute viral bronchiolitis and who received high-flow nasal cannulas upon admission to the pediatric intensive care unit. Patients were categorized into two groups, the Success Group and Failure Group, on the basis of high-flow nasal cannula therapy efficacy. The primary outcome was treatment failure, which was defined as the transition to invasive or noninvasive ventilation. The analyzed variables included age, sex, weight, high-flow nasal cannula parameters, vital signs, risk factors, comorbidities, and imaging. Acute viral bronchiolitis severity was assessed using the Wood-Downes Scale, and functional status was assessed via the Functional Status Scale, both of which were administered by trained physiotherapists.

**Results:**

In total, 162 infants with acute viral bronchiolitis used high-flow nasal cannulas, with 17.28% experiencing treatment failure. The significant differences between the Failure and Success Groups included age (p = 0.001), weight (p = 0.002), bronchiolitis severity (p = 0.004), initial high-flow nasal cannula flow (p = 0.001), and duration of use (p = 0.000). The cutoff values for initial flow (≤ 12L/min), weight (≤ 5kg), and Wood-Downes score (≥ 9 points) were determined from the ROC curves. Initial flow ≤ 12L/min was the most predictive for failure (AUC = 0.71; 95%CI: 0.61 - 0.84; p = 0.001). Multivariate analysis indicated that weight was a protective factor (RR = 0.87; 95%CI: 0.78 - 0.98), duration of use reduced the risk of failure (RR = 0.49; 95%CI: 0.38 - 0.64; p = 0.000), and Wood-Downes score was not significant (RR = 1.04; 95%CI: 0.95 - 1.14; p = 0.427). Weight explained 84.7% of the variation in initial flow.

**Conclusion:**

Risk factors for high-flow nasal cannula therapy failure in bronchiolitis patients include younger age, consequently lower weight, and a lower initial flow rate.

## INTRODUCTION

Acute viral bronchiolitis (AVB) is an inflammatory condition of the lower airways. Its pathophysiology involves airway resistance, muscle fatigue, hypoxemia, and alveolar atelectasis, leading to respiratory failure due to ineffective ventilation and perfusion.^(1)^Acute viral bronchiolitis is one of the leading causes of hospitalization in infants within the first 12 months of life, with admission rates ranging from 2 to 10%, and approximately 5 to 7% of these hospitalizations progress to ventilatory support.^(2,3)^

Traditional treatments for bronchiolitis have historically involved nutritional support and oxygen therapy.^(4)^ In this context, high-flow nasal cannula (HFNC) systems have emerged as innovative, widely accepted, alternative alternatives for critically ill patients of all ages.^(5)^

A high-flow nasal cannula represents a noninvasive ventilatory support method that delivers a gas flow, usually oxygen, that is appropriately humidified and heated. Its beneficial effects include reducing nasopharyngeal dead space, decreasing inspiratory resistance, providing an adjustable and stable concentration of oxygen (FiO_2_) according to the patient’s needs, and reducing metabolic expenditure for heating and humidifying inspired air. The simplicity and good patient adaptation of this approach are highlighted compared with those of traditional methods.^(6)^

Despite the growing interest in and application of HFNC systems in pediatrics, especially in cases of AVB, the effectiveness of these systems has not been comprehensively analyzed. In this context, the present study aims to fill gaps in the literature by identifying predictive factors for failure in the initial use of high-flow nasal cannulas in children diagnosed with AVB and under 24 months of age who are admitted to the pediatric intensive care unit (ICU).

## METHODS

The present study is characterized as a single-center retrospective cohort study involving infants < 24 months admitted to the pediatric ICU at *Hospital Moinhos de Vento* - Porto Alegre (RS), Brazil, from March 2018 to July 2023.

The study included patients aged less than 24 months who were diagnosed with AVB (ICD J21, J21.0, J21.8), classified as moderate or severe bronchiolitis, and received initial treatment with high-flow nasal cannulas in the pediatric ICU. Patients were categorized into two groups, the Success Group (SG) and Failure Group (FG), - based on HFNC therapy efficacy, and no patients were excluded.

**Figure 1:**
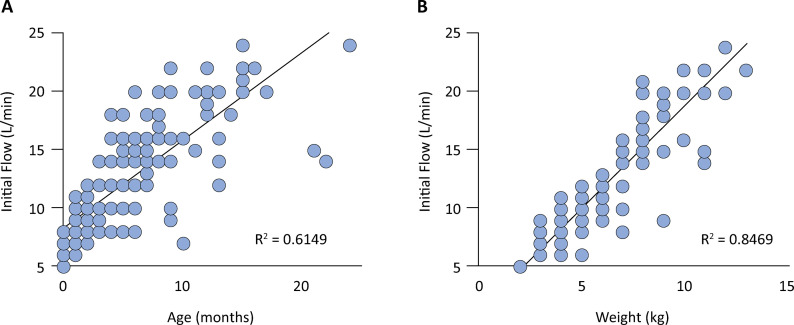
Relationships between the variables initial flow (L/min), weight (kg), and age (months).

The institutional protocol stipulates that patients classified with moderate bronchiolitis should be admitted to regular inpatient units and/or intensive care units, whereas severe cases should be admitted to the pediatric ICU (Figure 1S - Supplementary Material).

At the institution, the prescription of HFNC is assigned to the medical team based on clinical evaluation of the patient. Initial installation and parameter adjustments are conducted by the physiotherapy team, whereas maintenance and supervision of humidification and warming care are shared between the nursing and physiotherapy teams. According to the institutional protocol, the initial oxygen flow rate was set at 2L/kg/min. For children under 1 year old, the initial FiO_2_ was 40%, whereas for those over 1 year old, it begins at 60% FiO_2_. FiO_2_ is promptly adjusted to achieve a minimum target saturation of 92 - 94%, or higher based on the patient’s clinical response.

During the weaning process, the initial step involves reducing FiO_2_ to less than 40%, followed by gradual flow rate reduction until it reaches 1L/kg/min. This process is continuously monitored to ensure that the patient adequately responds to the reduction in oxygen support. The decision to discontinue HFNC therapy is made when the administration reaches 1L/kg/min, indicating a successful transition to reduced supplemental oxygen dependence.

The primary outcome, characterized by HFNC treatment failure, was defined as the escalation of therapy to invasive or noninvasive ventilation with continuous positive airway pressure, as recorded in the electronic medical records. Therapy failure was defined based on the clinical judgment of the attending physician or the on-call physician, without a preestablished protocol.

The variables analyzed were obtained from electronic records and included age, sex, weight, initial HFNC parameters (flow and FiO_2_), vital signs, risk factors (prematurity or bronchopulmonary dysplasia), comorbidities (cardiac/neurological diseases), imaging findings (X-ray), duration of HFNC use, length of stay in the ICU, and clinical outcome. Patients were allocated to the FG or SG for analysis, with records and evaluations centered on the time of therapy implementation.

Acute viral bronchiolitis was classified using the Wood-Downes scale (WD). This scale, which was originally developed to assess asthma severity, has also been applied in the pediatric context to evaluate bronchiolitis. The scale assesses wheezing, retractions, respiratory rate, heart rate, ventilation, and cyanosis, allowing the quantification of bronchiolitis severity in categories: mild (1 - 3), moderate (4 - 7), and severe (8 - 14)^(7)^(Table 1S - Supplementary Material). However, this scale was not used as part of an institutional protocol to designate the indication for the use of HFNC, noninvasive MV, or invasive MV and/or their escalation.

The profile of functional status was assessed by the Functional Status Scale (FSS). This scale, conceptualized based on daily living and adaptive behavior scales, is widely used to evaluate functional outcomes in hospitalized pediatric patients. The FSS is a free scale that consists of six domains (mental status, sensory function, communication, motor function, feeding, and respiratory status). Each domain is scored on a scale of 1 point (normal) to 5 points (very severe dysfunction). The total score ranges from 6 to 30 points, with lower scores indicating better functionality. The overall FSS score is categorized as follows: 6 - 7, adequate; 8 - 9, mild dysfunction; 10 - 15, moderate dysfunction; 16 - 21, severe dysfunction; and more than 21 points, very severe dysfunction^(8)^ (Table 2S - Supplementary Material).

The mentioned assessment scales are routinely used by the institution’s physical therapy service, with its practitioners being properly trained and qualified for their application. Other relevant variables are also frequently recorded in the electronic hospital system. However, the final decision regarding the escalation of oxygen therapy (invasive or noninvasive), although subject to multidisciplinary discussion, depends on the physician.

The research was submitted to the Ethics Committees of the institutions and is registered under numbers 4.498.847 and 4.582.915, CAAE 28507819.1.0000.5345 and 285078191.30.01.5330. Because this study was a retrospective, the requirement for informed consent was waived.

### Statistical analysis

Categorical variables were characterized through absolute and percentage frequencies. Numeric variables were analyzed for distribution and are presented as medians and interquartile ranges (IQRs). Normality was assessed using the K–S test, particularly for asymmetric distributions. The description was presented globally and stratified based on the occurrence or absence of failure in the use of HFNC.

To compare patient profiles with or without HFNC failure, hypothesis tests such as the χ2 test, Fisher’s exact test, and Mann–Whitney test were employed as appropriate. Spearman’s correlation coefficient was also used to examine correlations between quantitative predictor variables of HFNC failure.

In the multivariate approach, Poisson regression analysis with robust variance adjustment was employed, and two models, including WD scale points and usage time (days), were constructed. The first model included weight (kg), and the second model included initial flow (L/min). A multivariate model including weight and flow with age could not be created because of the high correlation (r > 0.8) among the three variables.

The relationships among the variables of age, weight, and flow are visually represented with scatter plots that show the coefficient of determination R^2^ derived from linear regression analysis. R^2^ is a metric that quantifies the proportion of variability in flow explained by age and weight.

A diagnostic accuracy assessment of the initial flow variables (L/min), weight (kg), and Wood-Downes (points) in predicting HFNC failure was conducted by constructing receiver operating characteristic (ROC) curves. The results included the area under the curve (AUC) with a 95% confidence interval (95%CI), sensitivity, specificity, and predictive values for the chosen cutoff points. In this study, curves were presented only for variables with significant areas.

All the statistical analyses were performed using SPSS (Statistical Package for Social Sciences) for Windows 22.0 (SPSS Inc., Chicago, IL, USA), with a significance level of 5% (p ≤ 0.05).

## RESULTS

In this study, 162 infants who were diagnosed with acute viral bronchiolitis and subjected to HFNC therapy were analyzed. Among these patients, 28 children (17.28%) experienced treatment failure. Specifically, 16 (57.1%) required escalation to noninvasive mechanical ventilation (MV), whereas 12 (42.8%) required invasive MV. No deaths were recorded in the analyzed sample (Table 1).

**Table 1:** General characteristics of patients overall and according to success or failure of high-flow nasal cannula therapy

Variable	Total (n = 162)	Group		p value
		Failure (n = 28)	Success (n = 134)	
Characteristics				
Sex				
Female	87 (53.7)	11 (39.3)	76 (56.7)	0.093 †
Male	75 (46.3)	17 (60.7)	58 (43.3)	
Age (months)	4 (2.0 - 7.00)	2 (1.0 - 3.5)	5 (2.0 - 8.0)	0.001 *
Weight (kg)	6.00 (4.0-8.0)	5.00 (4.0 - 6.0)	7.00 (4.0 - 8.0)	0.002 *
Initial assessments				
Wood-Downes score				
Moderate	105 (64.8)	11 (39.3)	94 (70.1)	0.004 †
Severe	57 (35.2)	17 (60.7)	40 (29.9)	
Points	6.0 (6.00 - 8.00)	9.0 (6.00 - 12.00)	6.0 (5.00 - 8.00)	<0.001 *
Functional Status Score @@(points) ‡	7.0 (7.0 - 9.0)	7.0 (7.0 - 10.0)	7.0 (7.0 - 9.0)	0.471 *
HFNC parameters				
Flow initial (L/min)	12.0 (8.00 - 16.00)	8.0 (7.5 -12.00)	12.0 (9.00-16.00)	0.001 *
FiO_2_ initial (%)	42.5 (40.00 -50.00)	43.5 (40.00 - 50.00)	42.5 (40.00 -50.00)	0.829 *
Vital signs				
HR initial (bpm)	154.0 (141.0 - 167.0)	156.0 (147.5 - 170.5)	152.5 (140.0 - 167.0)	0.426 *
RR initial (irpm)	53.0 (47.0 - 60.0)	51.5 (48.0 - 56.0)	54.0 (46.0 - 61.0)	0.442 *
SpO_2_ initial (%)	98.0 (96.0 - 100.0)	98.0 (95.5 - 100.0)	98.0 (96.0 - 100.0)	0.703 *
Risk factors				
Time of use (days)	4.0 (3.0 - 5.0)	1.0 (0.0 - 1.0)	4.0 (3.0 - 6.0)	0.000 *
Prematurity	17 (10.5)	5 (17.9)	12 (9)	0.178 †
Bronchopulmonary dysplasia	6 (3.7)	1 (3.6)	5 (3.7)	1.000 †
Comorbidities				
Cardiac diseases	8 (4.9)	2 (7.1)	6 (4.5)	0.628 †
Neurological diseases	5 (3.1)	1 (3.6)	4 (3)	1.000 †
Radiographic findings				
No change	57 (35.2)	7 (25)	50 (37.3)	0.215 †
Diffuse infiltrate	64 (39.5)	15 (53.6)	49 (36.6)	0.094 †
Hyperinflation	10 (6.2)	1 (3.6)	9 (6.7)	1.000 †
Atelectasis	50 (30.9)	9 (32.1)	41 (30.6)	0.872 †
Pleural effusion	3 (1.9)	1 (3.6)	2 (1.5)	0.436 †
Primary outcomes				
Noninvasive ventilation		16 (57.1)		
Invasive ventilation		12 (42.8)		
Secondary outcomes				
Time of use (days)	4.0 (3.0 - 5.0)	1.0 (0.0 - 1.0)	4.0 (3.0 - 6.0)	0.000 *
Length of stay (days)	9.0 (7.0 - 13.0)	17.0 (12.5 - 22.0)	8.5 (7.0 - 11.0)	0.000 *
Death	0 (0)	0 (0)	0 (0)	NO

HFNC - high-flow nasal cannula; FiO_2_ - fraction of inspired oxygen; HR - heart rate; RR - respiratory rate; SpO_2_ - peripheral oxygen saturation; cardiac diseases - congenital heart disease; neurological diseases - cerebral palsy; NO - no death. The results are expressed as n (%) or medians (interquartile ranges). * Mann–Whitney test; † p values for the χ2 test and Fisher’s exact test; ‡ n = 138.

Various analyzed variables significantly differed between the FG and SG. In terms of age, an average of 2 months was observed in the FG, whereas an average of 5 months was observed in the SG (p = 0.001). Patients in the FG had an average weight of 5kg, whereas those in the SG had an average weight of 7kg in SG (p = 0.002). Additionally, the severity of bronchiolitis-induced ventilatory dysfunction (measured by the WD scale) differed between groups; 60.7% of patients in the FG and 29.9% of patients in the SG received severe scores (p = 0.004); furthermore, point grading also significantly differed between groups, with a score of 9 points in the FG and a score of 6 points in the SG (p < 0.001). With respect to the initial therapy flow, the initial value was 8.0L/minin the FG, whereas it was 12.0L/min in the SG (p = 0.001). The duration of hospitalization among children in the FG group was twice the duration in the SG group, 17 days and 8 days, respectively (p = 0.000). Other nonsignificant variables are described in table 1.

The results of the univariate analysis are shown in table 2. For the multivariate analysis, two models were constructed to better analyze the data: one model for the adjusted weight (kg) with the WD points and usage time (days) and another model for the adjusted initial flow (L/min) with the WD points and usage time (days).

**Table 2:** Strength of association: univariate analysis

Variable	p value	RR	95%CI	
Age (months)	0.004	0.84	0.74	0.95
Time of use (days)	0.000	0.45	0.37	0.56
Weight (kg)	0.000	0.76	0.66	0.87
Weight ≤ kg	0.003	3.13	1.46	6.68
Initial flow (L/min)	0.001	0.85	0.78	0.94
Initial flow ≤ 12L/min	0.003	4.57	1.66	12.56
WD (points)	0.000	1.30	1.16	1.46
WD = Severe	0.003	2.85	1.43	5.66
WD ≥ 9 points	0.000	3.52	1.83	6.75

RR - relative risk; 95%CI - 95% confidence interval; WD - Wood-Downes. p value, relative risk, and 95% confidence interval for univariate analysis of various factors related to treatment failure.

In the multivariate analysis that considered WD scale points, weight (kg), and usage time (days) as variables, adding one point to the WD score increased the risk of failure by 4% (RR = 1.04; 95%CI 0.95 - 1.14; p = 0.427); however, this impact was not significant. The weight variable was a protective factor, resulting in a 13% reduction in the risk of failure for each kilogram increase (RR = 0.87; 95%CI 0.78 - 0.98). Additionally, each day of use reduced the risk of failure by 51% (RR = 0.49; 95%CI 0.38 - 0.64; p = 0.000) (Table 3).

**Table 3:** Multivariate analysis: model for weights adjusted by Wood-Downes points and usage time

	p valor	RR	95%CI	
WD (points)	0.427	1.04	0.95	1.14
Weight (kg)	0.020	0.87	0.78	0.98
Time of use (days)	0.000	0.49	0.38	0.64

RR - relative risk; 95%CI - 95% confidence interval. Multivariate analysis showing the model for weights adjusted by Wood–Downes points and usage time.

In the multivariate analysis model that considered WD points, initial flow (L/min), and usage time (days), each additional WD point increased the risk of failure by 4% (RR = 1.04; 95%CI 0.95 - 1.14; p = 0.427); however, this impact was not significant. However, a 6% reduction in risk was associated with each L/min increase in the initial flow (RR = 0.94; 95%CI 0.89 - 0.99; p = 0.032), whereas each additional day of use reduced the risk by 51% (RR = 0.49; 95%CI 0.38 - 0.64; p = 0.000) (Table 4).

**Table 4:** Multivariate analysis: model for initial flow adjusted by Wood-Downes points and usage time

	p valor	RR	95%CI	
WD (points)	0.427	1.04	0.95	1.14
Initial flow (L/min)	0.032	0.94	0.89	0.99
Time of use (days)	0.000	0.49	0.38	0.64

RR - relative risk; 95%CI - 95% confidence interval. Multivariate analysis showing the model for initial flow adjusted by the number of Wood-Downes points and usage time.

The decision was made to exclude the age variable in the multivariate analysis because of the high correlation (r > 0.8) with weight and flow. The presence of multicollinearity could distort coefficient estimates, impairing the interpretation of the results.

Weight was the most significant factor, explaining 84.7% of the variation in initial flow; age explained 61.5% of the variation, and time only explained 4.64% (Figure 1). The WD scale does not significantly correlate with the analyzed variable.

Weight was the most significant factor, explaining 84.7% of the variation in initial flow, whereas age explained 61.5%.

**Figure 2:**
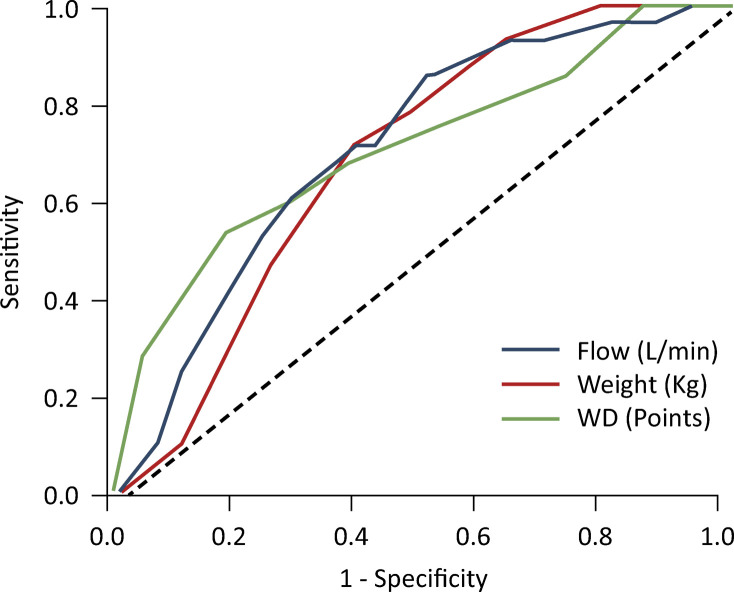
ROC curves for predicting high-flow nasal cannula failure using flow (L/min), weight (kg), and Wood-Downes (points)

The cutoff values of the parameters were determined through ROC curves (Figure 2). A cutoff value ≤ 12L/min for initial flow had an AUC of 0.71 (95%CI 0.61 - 0.84; p = 0.001), a sensitivity of 85.7%, and a specificity of 49.3%. The associated positive predictive value (PPV) was 26.1%, whereas the negative predictive value (NPV) reached 94.3%. A cutoff point ≤ 5kg for the weight (kg) variable had an AUC of 0.68 (95%CI 0.58 - 0.77; p = 0.002), a sensitivity of 71.4%, and a specificity of 61.2%. The associated PPV was 27.8%, whereas the NPV reached 91.1%. A threshold of ≥ 9 points for the WD (points) variable exhibited an AUC of 0.71 (95%CI 0.59 - 0.82; p = 0.001), a sensitivity of 53.6%, and a specificity of 81.3%. The associated PPV was 37.5%, whereas the NPV reached 89.3%.

The cutoff value of ≤ 12L/min for initial flow resulted in an area under the curve of 0.71 (95%CI 0.61 - 0.84; p = 0.001), with a sensitivity of 85.7% and specificity of 49.3%. The positive predictive value was 26.1%, and the negative predictive value was 94.3%. For the weight (kg) variable, with a cutoff point of ≤ 5kg, the area under the curve was 0.68 (95%CI 0.58 - 0.77; p = 0.002), the sensitivity was 71.4%, the specificity was 61.2%, the positive predictive value was 27.8%, and the negative predictive value was 91.1%. With respect to the Wood–Downes (points) variable, with a threshold of ≥ 9 points, the area under the curve was 0.71 (95%CI 0.59 - 0.82; p = 0.001), the sensitivity was 53.6%, and the specificity was 81.3%. The positive predictive value was 37.5%, and the negative predictive value reached 89.3%.

WD - Wood-Downes.

A comparison of the three variables reveals that the initial flow (≤ 12L/min) has a better discriminative capacity than the other two variables do. However, the WD variable (≥ 9 points) shows relatively high specificity (81.3%), indicating its potential in specific clinical contexts.

## DISCUSSION

Our findings highlight that risk factors for HFNC therapy failure in bronchiolitis patients include younger age, consequently lower weight, and a low initial flow rate.

The literature presents wide variation in high-flow therapy failure rates in bronchiolitis patients, ranging from 0% to 50%.^(9-12)^In this study of 162 infants who were diagnosed with AVB and treated with HFNC, 17.28% did not respond to treatment.

In our study, most HFNC failures occurred within a 24-hour period, with a median time to failure of 1 day. Studies have demonstrated that most failure cases can be predicted within 1 - 14 hours.^(13-15)^ This concordance between our findings and those of previous studies underscores the crucial importance of intensive monitoring and continuous monitoring during the initial phases of treatment.

We consistently observed that higher flow rates (≥ 12L/min) tend to be more beneficial, indicating that HFNC therapy may require a higher initial flow to be effective. Despite variations, most studies suggest initiating HFNC treatment with a flow rate of 2L/kg/min.^(16,17)^

Studies conducted by Ball et al.^(18)^and Papoff et al.^(19)^emphasize that initial flow rates play a crucial role in clinical outcomes, including improvements in ventilatory mechanics, reductions in respiratory effort, and treatment success rates. These recent data support our results, reinforcing the importance of this study in bringing cutoff points into its analyses.

A plausible explanation for the likelihood of therapy failure in children ≤ 5kg may be related to anatomophysiological factors combined with disease severity. In younger children, airways are naturally narrower, and the acute edema and inflammation caused by bronchiolitis further make tolerating and fully utilizing the flow provided by HFNC difficult. Diverging from our results, another study showed that HFNC therapy is more effective in children < 8kg.^(20)^

Another factor is that low-birth-weight children often have lower respiratory reserves, meaning that their lungs have less capacity to handle any type of respiratory stress.^(21)^ Additionally, predominantly nasal breathing (< 4 months) and the presence of excessive nasal secretions may impair flow delivery, which could hinder bronchiolitis treatment and lead to an inadequate response to therapy.

Identifying initial flow as a key indicator, with details on its significant relationship with failure risk and its high sensitivity, enriches the understanding of therapy dynamics in question. Furthermore, the influence of weight and age on initial flow variability evidenced by this study adds analytical depth to the findings. These insights have the potential to influence future treatment protocols and clinical practices, thus promoting a more effective and personalized approach to bronchiolitis management in pediatric patients.

This study revealed that every one-point increase on the Wood-Downes scale implies a 4% increase in therapeutic failure risk. These data highlight the practical relevance of these findings in specific clinical contexts. In line with our results, a retrospective study by Baquedano Lobera et al.^(22)^ also used the WD scale and described greater severity as a significant factor. The scarcity of studies using this scale as a failure predictor underscores the importance of our findings.

The literature addresses various predictors of failure in children receiving HFNC therapy, such as the respiratory rate and/or heart rate,^(16,23)^high Pediatric Risk of Mortality (PRISM III) score, ROX index,^(24)^high Pediatric Early Warning System (PEWS) score,^(25)^and the SpO_2_/FiO_2_ ratio.^(26)^Although we did not find significant data on physiological parameters, this study is the first to report specific cutoff points for initial flow and weight.

The study revealed a significant association between ineffective HFNC therapy and prolonged hospital stay, with the hospitalization time of nonresponsive patients being double that of responsive patients. Therefore, unsuccessful HFNC therapy may indicate that the disease condition is more severe and that these children likely need other forms of respiratory support or medical interventions, which in turn prolong their hospital stay.

This study is subject to the inherent limitations of a retrospective, single-center cohort study. At the institution, a protocol or rigorous method for declaring failure in HFNC therapy was not established. Importantly, the lack of longitudinal follow-up precluded the evaluation of variables at the time of therapy failure. Consequently, all analyzed data were obtained at the time of therapy implementation, which constitutes a significant limitation of this study. The sample size may have been impacted by the SARS-CoV-2 pandemic, which was initially associated with a reduction in respiratory infection diseases, such as bronchiolitis, due to reduced social interaction.^(27)^

However, the current study stands out for several reasons. First, the adopted methodology was rigorous and characterized by a detailed and statistically robust analysis of variables, which ensured the reliability of the results. Identifying predictive factors for HFNC treatment failure, highlighted during the initial stage of therapy, allows for more precise decisions, such as considering alternative interventions, intensifying care in cases of failure, and guiding therapy utilization protocols. Further prospective research and controlled studies are recommended to confirm these associations and establish the best monitoring and intervention criteria.

## CONCLUSION

Close monitoring of patients under high-flow nasal cannula therapy is essential because these patients are prone to requiring additional intensive care. Our research highlights that risk factors for high-flow nasal cannula therapy failure in bronchiolitis patients include younger age, consequently lower weight, and a lower initial offered flow.

## SUPPLEMENTARY MATERIAL

Predictive factors for high-flow nasal cannula failure in patients with acute viral bronchiolitis admitted to the pediatric intensive care unit


