# Supplementary material for: Predictive factors for high-flow nasal cannula failure in patients with acute viral bronchiolitis admitted to the pediatric intensive care unit
Source: Crit Care Sci. 2025 Feb 10;37:e20250161. doi: 10.62675/2965-2774.20250161 (PMC11869819; doi:10.62675/2965-2774.20250161)
Supplement: Supplementary file 1 [file 2965-2774-ccsci-37-e20250161-suppl.pdf]

Critical Care Science
https://doi.org/10.62675/2965-2774.20250161

SUPPLEMENTARY MATERIAL

Predictive factors for high-flow nasal cannula failure in patients with acute viral bronchiolitis admitted to the pediatric intensive care unit

Patrick Jacobsen Westphal[1], Cassiano Teixeira[2], João Ronaldo Mafalda Krauzer[1], Mirelle Hugo Bueno[1], Priscilla Alves Pereira[1], Sandro V. Hostyn[1], Marcela Doebber Vieira[1], Camila Durante[1], Cristiane Bündchen[2]

Table 1S - Wood-Downes Scale (Modified by Ferrés)

Description	0	1	2	3
Wheezing	None	End of expiration	Entire expiration	Inspiration + expiration
Retractions	None	Subcostal/lower intercostal	"1" + supraclavicular + nasal flaring	"2" + lower intercostal + suprasternal
Respiratory rate	< 30	31 - 45	- 60	> 60
Heart rate	< 120	> 120		
Ventilation	Good	Regular, symmetric	Decreased	Silent chest (absence of wheezing)
Cyanosis	Not present	Present		

Source: Ferrés Mataró J, Mangues Bafalluy MA, Farré Riba R, Juliá Brugues A, Bonal de Falgas J. Adrenalina subcutánea *versus* salbutamol inhalado en el tratamiento de la crisis asmática infantil. An Esp Pediatr .1987;27(1):37-40.
Scoring: Mild: 1 - 3 points; Moderate: 4 - 7 points; Severe: 8 - 14 points.

Table 2S - Functional Status Scale (FSS)

	Normal (Points = 1)	Mild dysfunction (Points = 2)	Moderate dysfunction (Points = 3)	Severe dysfunction (Points = 4)	Very severe dysfunction (Points = 5)
Mental state	Normal sleep/wake cycles; appropriate responsiveness	Sleepy but responsive to noise/touch/ movement; reduced social responsiveness	Lethargic and/or irritable	Minimal arousal to stimuli (stupor)	Non-responsive coma or vegetative state
Sensory functionality	Intact hearing and vision; responsive to touch	Suspected hearing or vision loss	Unresponsive to auditory or visual stimuli	Unresponsive to auditory or visual stimuli	Abnormal responses to pain or touch
Communication	Appropriate vocalization; interactive expressions	Reduced vocalization or social response	No attention-seeking behavior	No expression of discomfort	Absence of communication
Motor functioning	Coordinated movements; normal muscle control	Functional impairment in one limb	Functional impairment in two or more limbs	Poor head control	Diffuse spasticity, paralysis, or decerebrate/decorticate posturing
Feeding	All intake oral with appropriate assistance for age	Needs oral/tube feeding or age-inappropriate assistance	Oral and tube feeding	Parenteral nutrition with oral/tube feeding	Exclusive parenteral nutrition
Respiratory status	Ambient air; no artificial support or aids	Oxygen therapy and/or airway suctioning	Tracheostomy	CPAP for part/all of the day; partial mechanical support	Full-time mechanical ventilation

Source: Pollack MM, Holubkov R, Glass P, Dean JM, Meert KL, Zimmerman J, Anand KJ, Carcillo J, Newth CJ, Harrison R, Willson DF, Nicholson C; Eunice Kennedy Shriver National Institute of Child Health and Human Development Collaborative Pediatric Critical Care Research Network. Functional Status Scale: new pediatric outcome measure. Pediatrics. 2009;124(1):e18-28.
Scoring: 6 - 7 adequate; 8 - 9 mild dysfunction; 10 - 15 moderate dysfunction; 16 - 21 severe dysfunction; and more than 21 points, very severe dysfunction.
CPAP - continuous positive airway pressure.

This is an open access article under the CC BY license (https://creativecommons.org/licenses/by/4.0/).

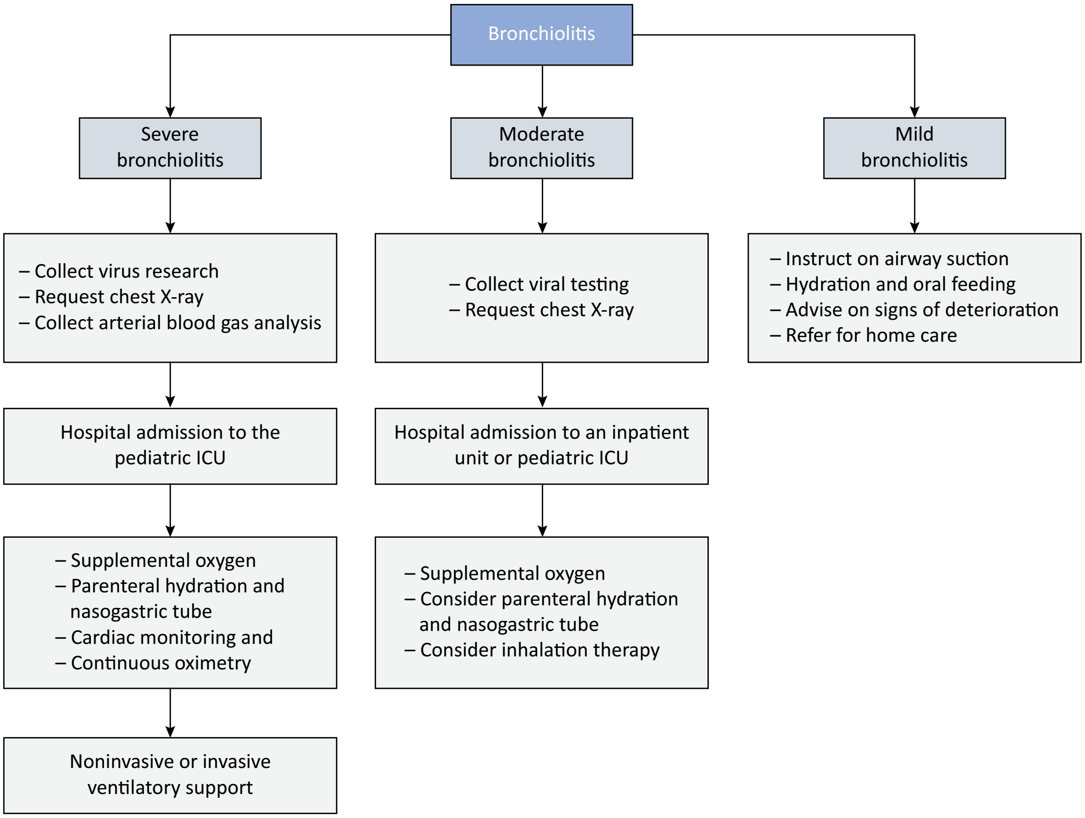

Figura 1S - Algorithm for bronchiolitis management - *Hospital Moinhos de Vento.*
ICU - intensive care unit.
